# Evaluation of interest trends among the general population and scientific community in migraine surgery

**DOI:** 10.1016/j.jpra.2026.03.001

**Published:** 2026-03-06

**Authors:** Edoardo Raposio, Andrea Antonini, Elisa Bertulla

**Affiliations:** a Plastic Surgery Chair, Department of Surgical Sciences and Integrated Diagnostics (DISC), University of Genova, Genova, Italy; b Plastic and Reconstructive Surgery Division, IRCCS Azienda Metropolitana Ospedaliera, Genova, Italy

**Keywords:** Migraine surgery, Headache surgery, Google Trends, Public interest, Infodemiology

## Abstract

**Background:**

Online search behavior reflects public curiosity, and Google Trends is increasingly used to assess interest in medical topics. Migraine surgery has emerged in recent decades as a promising option for patients with refractory headaches, drawing growing clinical and scientific attention.

**Objective:**

To analyze temporal trends in public interest in migraine surgery using Google Trends and compare them with scientific output indexed in PubMed.

**Methods:**

Worldwide Google Trends data for “Migraine Surgery” and “Headache Surgery” from 2004 to 2025 were aggregated annually. PubMed was searched for relevant publications in the same period. Descriptive statistics, Spearman correlation, and linear regression assessed associations between search interest and publication trends.

**Results:**

Searches for “Migraine Surgery” remained minimal until 2007, then increased steadily, for the remainder of the study period. PubMed publications began in 2010 and rose in parallel with public interest. For “Headache Surgery,” online searches also increased after 2007, but publication numbers fluctuated, showing a weaker relationship. Spearman correlation was strong for “Migraine Surgery” (ρ = 0.904) and moderate for “Headache Surgery” (ρ = 0.490). Regression analysis confirmed these patterns: the model for “Migraine Surgery” was significant (*F* = 17.83, *p* < 0.001; R² = 0.48), whereas the model for “Headache Surgery” was not (*F* = 3.19, *p* = 0.090; R² = 0.14).

**Conclusions:**

Public interest in migraine surgery closely parallels scientific activity, while the broader term “Headache Surgery” shows weaker associations, probably due to the less specific terminology. Integrating digital epidemiology with bibliometric analysis may help anticipate patient needs and guide healthcare planning.

Level of evidence: V

## Introduction

Internet search behavior reflects population-level curiosity, concern, and intent. Health researchers increasingly using search data (so-called “infodemiology”) to identify seasonal patterns, responses to public announcements, and unmet patient needs.^1^^,^^2^

Google Trends (Googleplex, Mountain View, CA, USA) is the most widely public tool used for analyzing online behavior, offering reproducible relative search volume (RSV) data directly from Google.^3^

Google Trends has been increasingly used in medicine to investigate public interest, seasonal patterns, and responses to health-related announcements across a variety of fields.^4^, ^5^, ^6^, ^7^, ^8^

In plastic surgery it has been applied to study interest in specific procedures, including breast augmentation, facial cosmetic procedures, gender-affirmation surgery, cosmetic body surgery, peripheral nerve surgery^9^, ^10^, ^11^, ^12^, ^13^ demonstrated moderate to strong correlation to surgical volume activity. Supporting this, a recent systematic review by Bellaire et al.^14^ recognized Google Trends as an emerging and valuable tool in plastic surgery, outlining its strengths, limitations, and best practices for the use of keywords, regions, time periods, and categories.

Migraine surgery has emerged as a novel intervention for patients with refractory headache, attracting growing clinical and scientific interest over the past 2 decades.^15^, ^16^, ^17^, ^18^, ^19^, ^20^, ^21^, ^22^, ^23^, ^24^, ^25^, ^26^, ^27^, ^28^, ^29^

We therefore thought it would be of interest to evaluate the trends of interest from the general public regarding migraine surgery over the last 2 decades using the aforementioned software, and to compare the obtained results with the scientific research trends via PubMed (National Library of Medicine, Bethesda, MD, USA) to explore potential links or patterns between public interest and scientific output, as no previous studies have directly assessed this relationship.

Understanding differences between data obtained by this tool may provide valuable insights for researchers studying patient interest in migraine surgery and for physicians monitoring referral demand.

## Methods

We conducted a Google Trends search for the keywords “Migraine Surgery” and “Headache Surgery” using the filters “Worldwide” and “All categories,” covering the period from January 1, 2004, to September 10, 2025. The search starts in 2004 because this was the earliest date with available Google Trends data.

Since Google Trends provides monthly data, the monthly scores were averaged for each year to obtain an annual mean score. Consequently, brief monthly peaks may be smoothed out in the annual average.

Google Trends provides normalized Relative Search Volume (RSV) scaled from 0 (minimal popularity) to 100 (maximum popularity) in relation to the peak during the selected time range and geography. Although it does not reveal absolute daily search counts, RSV is particularly useful for comparing periods of low and high public interest.

The same search was then performed on PubMed. The annual publications counts were downloaded and entered into an Excel (Microsoft Corporation, Redmond, WA, USA) chart. Article titles and abstracts were subsequently reviewed for relevance to ensure all included studies specifically addressed migraine surgery. Because PubMed data are available only at annual resolution, it was not possible to assess seasonal patterns in scientific output.

Statistical analysis (Spearman correlation and linear regression analysis) was performed using Microsoft Excel.

## Results

To explore temporal patterns, we analyzed Google Trends search volumes for migraine surgery alongside the number of related publications indexed in PubMed. Subsequent analyses focused on discovering potential associations between public interest and scientific output.

Google Trends RSV and PubMed counts is shown in Table 1.

**Table 1:** Google Trends RSV (average for each month) and PubMed counts.

Year	Migraine surgery Google Trends RSV	Migraine surgery Pubmed counts	Headache surgery Google Trends RSV	Headache surgery Pubmed counts
2004	8,3	0	7,41	7
2005	6,4	0	0	18
2006	11,5	0	11,3	6
2007	9,75	0	20	16
2008	17,25	0	24,1	18
2009	23,58	0	27,9	2
2010	25,66	1	30	22
2011	32,66	2	31,9	25
2012	31,5	5	38,3	22
2013	33	4	42,3	29
2014	30,6	5	42	22
2015	28,41	5	45	19
2016	30,1	6	46,1	3
2017	34,2	9	50,41	28
2018	35,4	9	54,3	23
2019	36,9	12	57,5	33
2020	33,58	8	53,5	22
2021	36,1	12	58	21
2022	37,6	16	59,5	25
2023	38,4	17	63,7	26
2024	38,6	15	66,8	24
2025	53	6	74,3	13

Analysis of Google Trends data shows (Figure 1a) that global search interest for keyword “migraine surgery” was low and relatively stable from 2004 until around 2007, with only minor fluctuations. Starting in 2008, interest began to rise noticeably, remaining steady until a modest increase around 2018. After stabilizing again through 2024, the search interest reached the highest recorded value in 2025, the final year of observation.

**Figure 1a:**
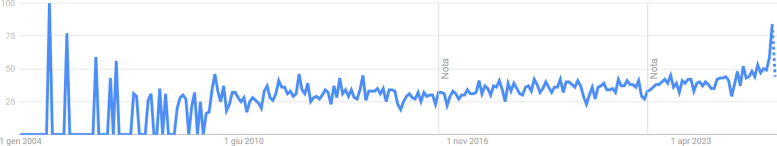
Temporal trends of “Migraine Surgery” search on Google Trends.

The analysis of Google Trends by countries (Figure 1b) shows that the countries with the highest public interest were UK and USA, followed by Ireland, Lebanon and Australia.

**Figure 1b:**
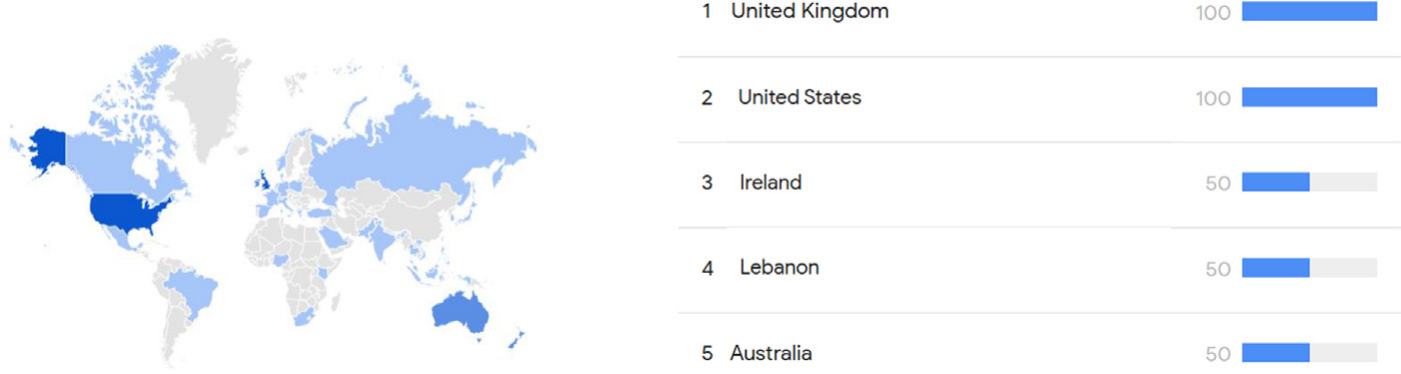
Top region searching for “Migraine Surgery” on Google Trends.

The PubMed query for “Migraine Surgery” yielded no publications until 2010, followed by a limited number until 2011. Between 2012 and 2016, 4 and 5 publications per year were retrieved. A notable increase began in 2017, culminating in a peak of 17 publications in 2024, before declining to 6 in 2025.

Analysis of Google Trends data shows (Figure 2a) that global search interest for keyword “Headache Surgery” was low and relatively stable in 2004, 2005 and 2006. Starting in 2007, interest began to rise, continuing to increase over subsequent years with only minor fluctuations.

**Figure 2a:**
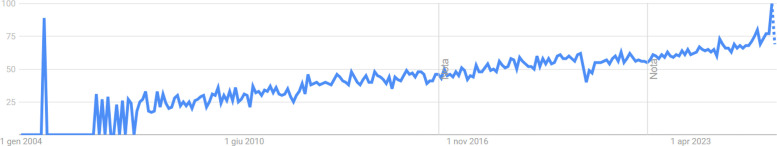
Temporal trends of “Headache Surgery” search on Google Trends.

The analysis of Google Trends by countries (Figure 2b) shows that the countries with the highest public interest were UK and USA, followed by Kenya, Australia and Ireland.

**Figure 2b:**
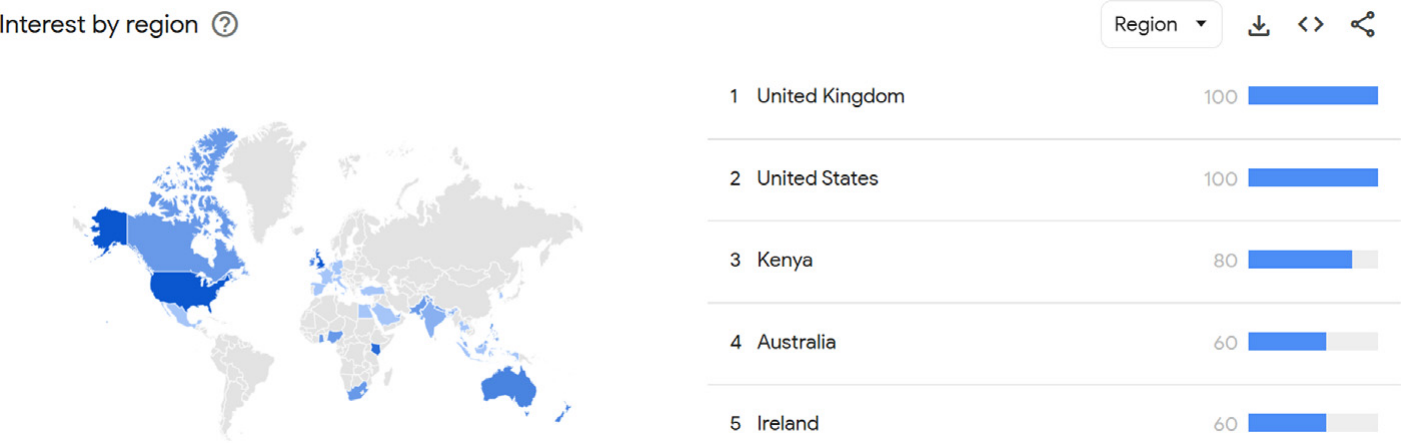
Top region searching for “Headache Surgery” on Google Trends.

The PubMed query for “Headache Surgery” showed substantial fluctuations. Early years (2004–2008) had generally moderate and variable counts, ranging from 6 to 18 publications per year. From 2010 onward, publication counts increased overall with occasional decreases, reaching a maximum of 33 records. In the most recent years (2020–2025), numbers stabilized with moderate variation, ranging from 13 to 26 per year.

**Figure 3:**
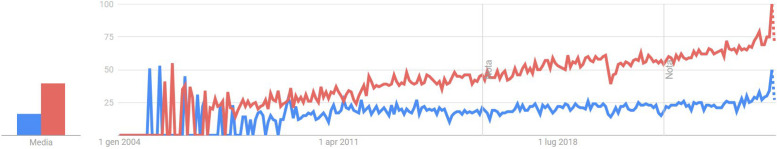
Comparison of the two keywords search on Google Trends. In blue “Migraine Surgery,” in red “Headache Surgery.”

In terms of Google Trends data, the search interest for both keywords is generally similar, although ‘headache’ shows slightly higher values, as it showed in Figure 3.

Temporal trends in both public and scientific attention are plotted in Figure 4a, Figure 4b.

**Figure 4a:**
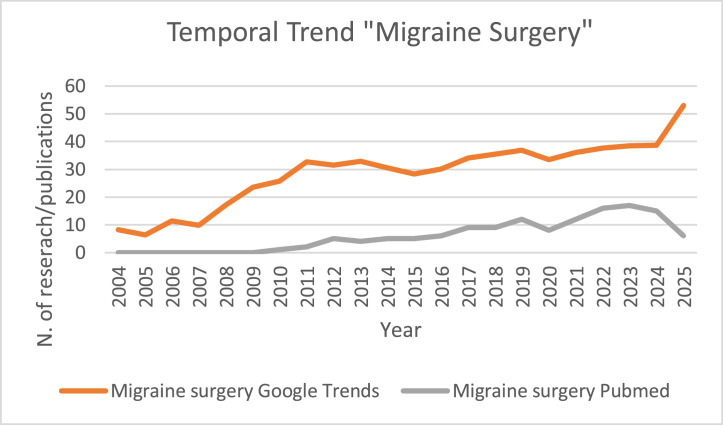
Line chart showing yearly trends (X axis = Year) of Google Trends interest (Y₁) and PubMed publications (Y₂) for keyword “Migraine Surgery.”

**Figure 4b:**
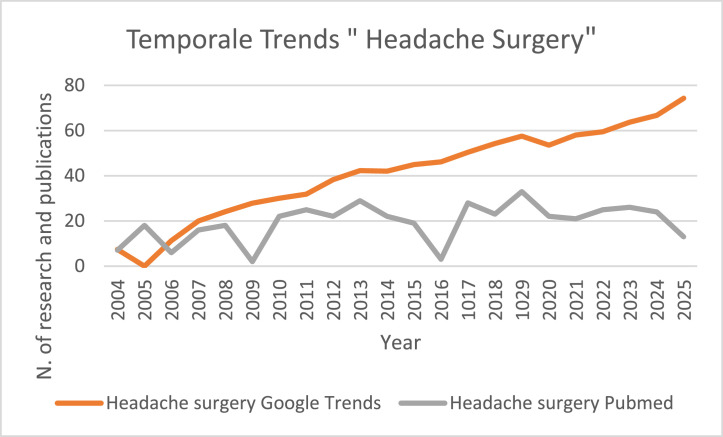
Line chart showing yearly trends (X axis = Year) of Google Trends interest (Y₁) and PubMed publications (Y₂) for keyword “Headache Surgery.”

The datasets were processed and analyzed to generate descriptive statistics and visual outputs for comparison across time.

The first step of the analysis was focused on descriptive statistics for the annual RSV and yearly PubMed publication counts, including measures of central tendency (mean, median), dispersion (standard deviation, variance), and distribution shape (skewness, kurtosis).

The data are summarized in Table 2.

**Table 2:** Descriptive statistics of Google Trends and PubMed data.

Type of analysis	Google Trends migraine surgery	Pubmed migraine surgery	Google Trends headache surgery	PubMed headache surgery
Mean	28,75	6	41,10	19,27
Median	32,08	5	43,65	22
Standard deviation	11,680910	5,6061191	20,071493	84,017108
Minimum	6,4	0	0	
Maximum	38,6	17	74,3	33
Variance	136,4436	31,42857	402,86483	70,588744
Skewness	−0,490096	0,6118314	−0,435721	−0,780706
Kurtosis	0,0706123	−0,7438086	−0,5478182	−0,046885

Given the skewed distributions and presence of outliers, non-parametric correlation was performed to further explore potential associations.

Spearman’s rank correlation showed a strong positive association between annual RSV values and yearly PubMed publication counts for the keyword “Migraine Surgery” (ρ = 0.904), and a moderate positive association (ρ = 0.490) for the keyword “Headache Surgery.”

Then, two separate regression analysis were conducted.

The first model about “Migraine Surgery” keyword (Figure 5a) was significant (*F* = 17.83, *p* < 0.001), with a coefficient of determination *R*^2^ = 0.48, indicating that approximately 48% of the variance in publication counts is explained by search trends. The regression coefficient for Google Trends was positive and significant (*b* = 0.352, *p* < 0.001).

**Figure 5a:**
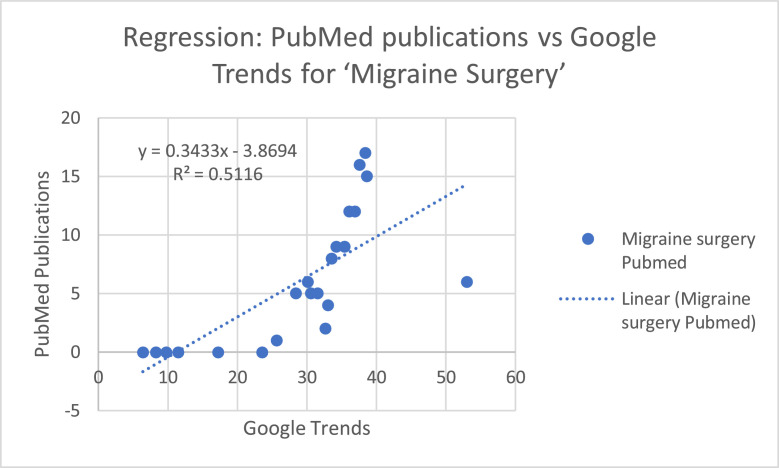
Regression model for “Migraine Surgery” keyword.

The regression model for “Headache Surgery” keyword (Figure 5b.) was not significant (*F* = 3.19, *p* = 0.090), with a coefficient of determination R² = 0.14, indicating that approximately 14% of the variance in publication counts is explained by search trends. The regression coefficient for Google Trends was positive but not significant (*b* = 0.162, *p* = 0.090).

**Figure 5b:**
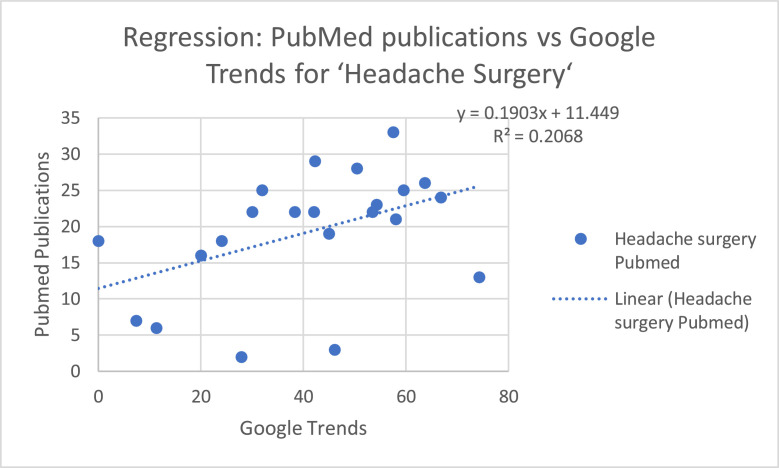
Regression model for “Headache Surgery” keyword.

## Discussion

Although it took some time to become widely recognized, migraine surgery has developed into a novel intervention for patients with refractory headache, generating growing interest among clinicians and researchers over the past 20 years.^25^^,^^27^^,^^30^, ^31^, ^32^, ^33^, ^34^, ^35^, ^36^, ^37^, ^38^, ^39^

This study provides a combined analysis of global online search interest and scientific publication trends for the keywords “Migraine Surgery” and “Headache Surgery” over the past 2 decades. Public interest, as measured by Google Trends, has generally increased for both keywords, although the patterns differ. For “Migraine Surgery,” search interest was low and stable until 2007, followed by a steady rise, peaking around 2018 and reaching the highest level in 2025. Publication counts on PubMed appeared in 2010 and gradually increased, peaking at 17 publications in 2024 before slightly declining in 2025.

For “Headache Surgery,” search interest was slightly higher overall, showing a progressive increase from 2007 onward. Publication trends were more variable, with moderate fluctuations in the early years and a general upward trend from 2010, reaching a maximum of 33 publications and stabilizing in recent years (2020–2025).

Spearman’s rank correlation revealed a strong positive association between RSV values and publication counts for “Migraine Surgery” (ρ = 0.904) and a moderate positive association for “Headache Surgery” (ρ = 0.490). Regression analysis confirmed these patterns: the model for “Migraine Surgery” was significant (*F* = 17.83, *p* < 0.001) with R² = 0.48, and the regression coefficient for Google Trends was positive and significant (*b* = 0.352, *p* < 0.001). In contrast, the model for “Headache Surgery” was not significant (*F* = 3.19, *p* = 0.090) with R² = 0.14, and the regression coefficient for Google Trends was positive but not significant (*b* = 0.162, *p* = 0.090).

The stronger association observed for “Migraine Surgery” may reflect increasing recognition of this procedure among both clinicians and patient communities. In contrast, the more moderate relationship for “Headache Surgery” may be due to the less specific terminology which may include a wider variety of searches and interests.

Overall, these results suggest that public interest may play a role in shaping scientific activity, especially for topics with higher visibility or perceived as clinically important.

There is an ongoing debate about the most appropriate terminology for surgical interventions targeting migraine and related headache disorders. Although the term “Migraine Surgery” has traditionally been used, some authors have suggested the broader term “Headache Surgery” to better capture the range of headache conditions that may be treated with these procedures. In this context, our findings may add to the discussion, as search interest increasingly favored “Headache Surgery,” while interest in “Migraine Surgery” appeared to level off. This trend may reflect a preference in public perception and information-seeking behavior for the broader terminology and could be viewed as lending support to the use of the term “Headache Surgery” rather than “Migraine Surgery.”

Few previous studies have compared public interest and scientific publications over time in other medical fields. Most reported generally parallel increases between the two but did not calculate formal correlations^8^^,^^40^, ^41^, ^42^; one study, however, did report a statistical association between public interest and scientific publications,^7^ highlighting the relative scarcity of such analyses and the novelty of ours. Although no prior studies have directly compared Google search trends with PubMed publication counts for migraine surgery, a recent analysis by Bishay et al.^43^ investigated public interest in migraine surgery and two migraine medications using Google Trends, showing that there are peaks of interest for medications related to approval and marketing events, whereas interest in migraine surgery remains constant over time, suggesting limited public awareness of this option.

This study has some limitations. Google Trends provides only normalized relative search volume rather than absolute counts, and its internal normalization algorithm is not fully transparent. The platform also lacks demographic stratifications, preventing age- or sex-specific analyses. It is important to note that Google Trends data are influenced by the language of the search query. When a keyword is specified in English, the resulting data predominantly reflect searches conducted using that specific term. Consequently, in countries where the primary language differs, observed search volumes may underestimate actual interest, as equivalent queries in the local language are not captured. PubMed indexing may not capture all relevant publications, particularly those from non-indexed journals or regional databases. Additionally, publication counts are available only at annual resolution, while Google Trends data are monthly; aggregation to yearly data prevented assessment of seasonal patterns or short-term fluctuations. Potential temporal delays between public interest and publication activity were not assessed, and Google Trends reflects the general internet-using population, whereas PubMed represents scientific productivity, two populations driven by different dynamics that may explain the weaker association observed for “Headache Surgery.”

Another limitation to consider is the potential temporal lag between trends observed in Google Trends and the publication of articles in databases such as PubMed. Scientific publications require time for data collection, manuscript preparation, and publication. As a result, peaks in interest reflected in search trends may precede or follow the appearance of corresponding literature. For future studies, it is therefore recommended to account for these temporal delays when comparing search data with published research, as doing so could improve the accuracy and interpretability of such analyses.

Even with these limitations, looking at public interest alongside scientific publications offers useful insights into how public curiosity and research priorities may be connected. Our findings also underscore the value of combining bibliometric analyses with digital epidemiology tools, such as Google Trends, to track evolving trends in medical topics. Such integrative approaches can help identify areas gaining clinical and scientific attention, potentially guiding research priorities, resource allocation, and patient education strategies. Future research could incorporate additional data sources, such as social media or clinical registries, and examine geographic or demographic variations to better understand the dynamics between public interest and scientific production. Overall, this study highlights the connection between public awareness, the ways people search for health information online, and scientific activity in the field of headache and migraine surgery, providing a framework for future investigations.

## Conclusions

Public interest in migraine and headache surgery, as measured by Google Trends, generally reflects scientific publication trends, with a stronger association observed for “Migraine Surgery.” These findings suggest that the curiosity of general public may influence or coincide with research activity, particularly for newly developed treatments. The weaker connection for “Headache Surgery” may be due to the less specific terminology which may capture a wider range of searches, reducing the measured correlation with scientific output. Integrating digital epidemiology tools with bibliometric analyses offer a useful approach to track patient interest, anticipate research needs, and guide healthcare planning. Overall, this study demonstrates the value of combining online search data with scientific output to better understand how public interest and research evolve together in surgical fields.

## Availability of data and materials

Data/materials are available from the corresponding author upon request.

## Author contributions

**ER** designed the study, supervisioned the whole project. **AA** co-performed the study. **EB** co-performed the study and wrote the manuscript. All authors contributed to editorial changes in the manuscript. All authors read and approved the final manuscript.

## Ethics approval and consent to participate

This article does not contain any studies with human participants or animals performed by any of the authors.

## Funding

None.

## Declaration of competing interest

The authors declare no conflict of interest.

## References

[1] Eysenbach G., Köhler C. (2002). How do consumers search for and appraise health information on the world wide web? Qualitative study using focus groups, usability tests, and in-depth interviews. BMJ.

[2] Carneiro H.A., Mylonakis E. (2009). Google trends: a web-based tool for real-time surveillance of disease outbreaks. Clin Infect Dis.

[3] Google. Google Trends. https://trends.google.it/trends/explore?geo=US&hl=it. Accessed January 2026.

[4] Cervellin G., Comelli I., Lippi G. (2017). Is Google Trends a reliable tool for digital epidemiology? Insights from different clinical settings. J Epidemiol Glob Health.

[5] Mavragani A., Ochoa G., Tsagarakis K.P. (2018). Assessing the methods, tools, and statistical approaches in Google Trends research: systematic review. J Med Internet Res.

[6] Arora V.S., McKee M., Stuckler D. (2019). Google Trends: opportunities and limitations in health and health policy research. Health Policy (New York).

[7] Laubach L., Chiang B., Sharma V., Jacobs J., Krumme J.W., Kuester V. (2023). Alternative and adjunct treatments for scoliosis: a Google Trends analysis of public popularity compared with scientific literature. Cureus.

[8] Pathak N., Radford Z.J., Kahan J.B., Grauer J.N., Rubin L.E. (2022). Publication frequency and Google Trends analysis of popular alternative treatments to arthritis. Arthroplast Today.

[9] Wilson S.C., Daar D.A., Sinno S., Levine S.M. (2018). Public interest in breast augmentation: analysis and implications of Google Trends data. Aesthetic Plast Surg.

[10] Tijerina J.D., Morrison S.D., Nolan I.T., Vail D.G., Nazerali R., Lee G.K. (2019). Google Trends as a tool for evaluating public interest in facial cosmetic procedures. Aesthet Surg J.

[11] Merrick E., Weissman J.P., Ascha M., Jordan S.W., Ellis M. (2022). National Trends in gender-affirming surgical procedures: a Google Trends analysis. Cureus.

[12] Duggan R.P., Tran J.P., Phillips L.G. (2020). Interest in plastic surgery during COVID-19 Pandemic: a Google Trends analysis. Plast Reconstr Surg Glob Open.

[13] Orlando N.A., Qiu C.S., ElNemer W., Tuffaha S.H. (2023). Google Trends analysis of peripheral nerve disease and surgery. World Neurosurg.

[14] Bellaire C.P., Rutland J.W., Sayegh F., Pesce R.R., Tijerina J.D., Taub P.J. (2021). Going viral: a systematic review of Google Trends in plastic surgery and a recommended framework for its use. Aesthet Surg J.

[15] Raposio G., Raposio E. (2022). Principles and techniques of migraine surgery. Eur Rev Med Pharmacol Sci.

[16] Raposio E., Raposio G. (2023). Surgical therapy of migraine: a 12-year single-center experience. Eur J Plast Surg.

[17] Raposio G., Baldelli I., Raposio E. (2024). Surgical therapy for primary headaches: techniques and results. Chirurgia (Bucur).

[18] Raposio E., Lago G., Fante C., Sanese G., Bertozzi N., Simonacci F. (2019). Minimally invasive Migraine Surgery: our 9-year experience. Plast Reconstr Surg Glob Open.

[19] Baldelli I., Mangialardi M.L., Salgarello M., Raposio E. (2020). Peripheral occipital nerve decompression surgery in migraine headache. Plast Reconstr Surg Glob Open.

[20] Raposio G., Raposio E. (2022). Surgical therapy of occipital (Arnold) neuralgia: a case series. Ann Med Surg (Lond).

[21] Raposio G., Raposio E. (2022). Temporal surgery for chronic migraine treatment: a minimally-invasive perspective. Ann Med Surg (Lond).

[22] Janis J.E., Barker J.C., Javadi C., Ducic I., Hagan R., Guyuron B. (2014). A review of current evidence in the surgical treatment of migraine headaches. Plast Reconstr Surg.

[23] Gfrerer L., Austen W.G., Janis J.E. (2019). Migraine surgery. Plast Reconstr Surg Glob Open.

[24] Gfrerer L., Austen W.G., Afifi A.M., Peled Z.M., Janis J.E. (2020). Surgical Treatment of Chronic Headaches and Migraines.

[25] Gfrerer L., Guyuron B. (2017). Surgical treatment of migraine headaches. Acta Neurol Belg.

[26] Guyuron B. (2021). The evolution of migraine surgery: two decades of continual research. My current thoughts. Plast Reconstr Surg.

[27] Guyuron B., Kriegler J.S., Davis J., Amini S.B. (2011). Five-year outcome of surgical treatment of migraine headaches. Plast Reconstr Surg.

[28] Guyuron B., Nahabet E., Khansa I., Reed D., Janis J.E. (2015). The current means for detection of migraine headache trigger sites. Plast Reconstr Surg.

[29] Guyuron B., Kriegler J.S., Davis J., Amini S.B. (2005). Comprehensive surgical treatment of migraine headaches. Plast Reconstr Surg.

[30] Raposio E., Caruana G. (2017). Tips for the surgical treatment of occipital nerve-triggered headaches. Eur J Plast Surg.

[31] Raposio E., Raposio G., Del Duchetto D., Tagliatti E., Cortese K. (2022). Morphologic vascular anomalies detected during migraine surgery. J Plast Reconstr Aesthet Surg.

[32] Raposio G., Antonini A., Gualdi A., Raposio E. (2023). Frontal site surgery for chronic migraine therapy. Acta Biomed.

[33] Baldelli I., Mangialardi M.L., Raposio E. (2020). Site V surgery for temporal migraine headaches. Plast Reconstr Surg Glob Open.

[34] Baldelli I., Zaottini F., Picasso R. (2025). Preoperative ultrasound evaluation for decompressive surgery for occipital neuralgia: preliminary results of a case-control study. J Plast Reconstr Aesthet Surg.

[35] Raposio E., Raposio G., Baldelli I., Peled Z. (2024). Active occipital motion with digipressure as preoperative screening in migraine surgery. Plast Reconstr Surg Glob Open.

[36] Chen G., You H., Juha H. (2021). Trigger areas nerve decompression for refractory chronic migraine. Clin Neurol Neurosurg.

[37] Raposio E. (2025). Temporal migraine surgery: relevance of local blood vessels in surgical technique. J Pioneer Med Sci.

[38] Gfrerer L., Raposio E., Ortiz R., Austen W.G. (2018). Surgical treatment of migraine headache: back to the future. Plast Reconstr Surg.

[39] Burstein R., Noseda R., Borsook D. (2015). Migraine: multiple processes, complex pathophysiology. J Neurosci.

[40] Cornelis F.H., Bokhari R., Geevarghese R., Filippiadis D.K. (2025). Cryoneurolysis: parallel growth in PubMed publications and Google Trends analysis reveals exponential interest. Cardiovasc Intervent Radiol.

[41] Hoffman A.F., Laspro M., Verzella A.N., Tran D.L., Rodriguez E.D. (2024). Facial masculinization surgery: an analysis of interest trends using search term analysis. Ann Plast Surg.

[42] Jahangiri Y., Gabr A., Huber T.C., Bochnakova T., Farsad K. (2023). Uterine fibroid embolization or myomectomy: how much marketing is enough? Comparative analysis of public search trends in Google and medical publications in PubMed. J Vasc Interv Radiol.

[43] Bishay A.E., Fijany A.J., Holan C. (2024). Analyzing Google search trends for migraine surgery and Nurtec in response to public announcements. Plast Reconstr Surg Glob Open.

